# Transcriptomic Signatures in Seeds of Apple (*Malus domestica* L. Borkh) during Fruitlet Abscission

**DOI:** 10.1371/journal.pone.0120503

**Published:** 2015-03-17

**Authors:** Sergio Ferrero, Lorenzo Carretero-Paulet, Marta Adelina Mendes, Alessandro Botton, Giulia Eccher, Simona Masiero, Lucia Colombo

**Affiliations:** 1 Dipartimento di BioScienze, Università degli Studi di Milano, Milan, Italy; 2 Department of Biological Sciences, University at Buffalo, Buffalo, New York, United States of America; 3 Department of Agronomy, Food, Natural Resources, Animals, and Environment, University of Padova, Agripolis, Legnaro, Italy; Chinese Academy of Sciences, CHINA

## Abstract

Abscission is the regulated process of detachment of an organ from a plant. In apple the abscission of fruits occurs during their early development to control the fruit load depending on the nutritional state of the plant. In order to control production and obtain fruits with optimal market qualities, the horticultural procedure of thinning is performed to further reduce the number of fruitlets. In this study we have conducted a transcriptomic profiling of seeds from two different types of fruitlets, according to size and position in the fruit cluster. Transcriptomic profiles of central and lateral fruit seeds were obtained by RNAseq. Comparative analysis was performed by the functional categorization of differentially expressed genes by means of Gene Ontology (GO) annotation of the apple genome. Our results revealed the overexpression of genes involved in responses to stress, hormone biosynthesis and also the response and/or transport of auxin and ethylene. A smaller set of genes, mainly related to ion transport and homeostasis, were found to be down-regulated. The transcriptome characterization described in this manuscript contributes to unravelling the molecular mechanisms and pathways involved in the physiological abscission of apple fruits and suggests a role for seeds in this process.

## Introduction

Apple (*Malus* x *domestica* L. Borkh) is one of the most cultivated fruit crops around the world and is becoming a model species since the recent elucidation of its genome sequence [1]. Similarly to many other fruit crops, apple trees develop an abundance of flowers that results in an excessive fruit load with a subsequent reduction in fruit quality. Many fruit tree species have developed a system of regulation of the fruit load whereby part of it is shed during early fruit development. While in some cases abscission of fruits is sufficient for regulating the fruit load, as in some species of *Citrus* [2], in apple trees physiological shedding reduces the number of fruits insufficiently [3–5].

**Fig 1:**
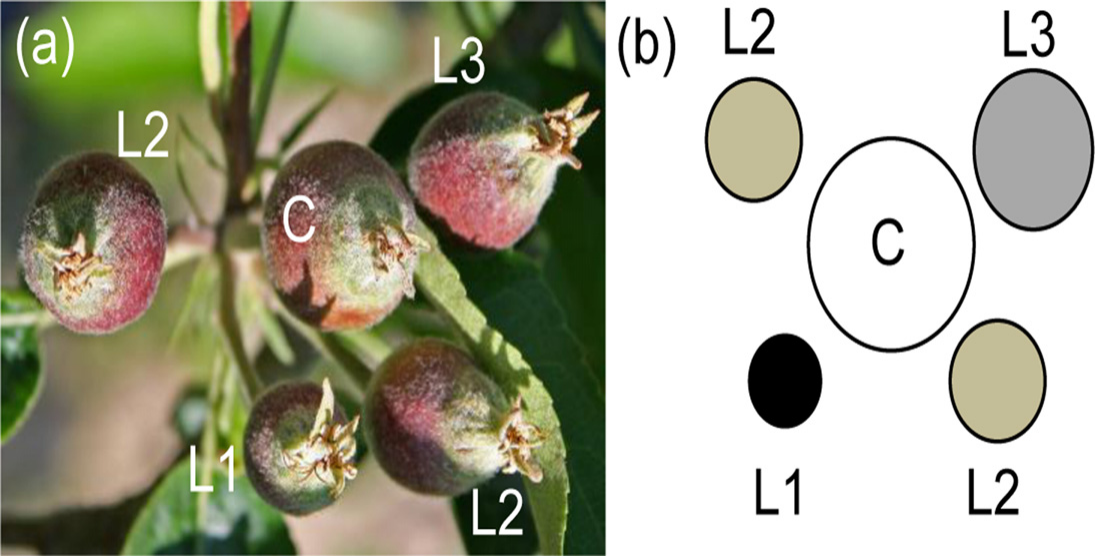
Apple cluster and abscission potential. (a) central fruit (C) is clearly bigger than lateral fruits (L), and (b) schematic representation of the abscission potential of apple fruitlets inside a cluster. Lateral fruits are classified by size (L3 > L2 > L1). Darker colors represent higher abscission potentials. (adapted from Botton *et al*., 2011).

In apples, the corymb is formed by 5–6 flowers [5]. The central flower usually blooms 1–3 days before the lateral flowers. Central fruits are thus bigger and more developed than the lateral ones. Among the lateral fruits a hierarchy also exists, and as a result different fruit dimensions can be found depending on the time of the flower blooming event (L3 > L2 > L1) (Fig. 1). Within the cluster, the positions and sizes of the fruits confer them different abscission potentials, by virtue of a phenomenon called correlative dominance [6] (Fig. 1b). Correlative dominance, also called correlative driven abscission (CDA), is a process controlled by the synthesis of auxin which is produced mainly in the seeds and is transported along the pedicel (polar auxin transport; PAT). The positions of the fruits inside the cluster determine the flux of auxin due to differences in the time of fertilization (fruit set) and the number of seeds in each fruit and this influences their abscission potentials [7–12]. In general, smaller lateral fruits have the highest probability of being shed [3]. The mechanism controlling CDA has to be distinguished from the senescence and abscission of ripe fruits [6]. A horticultural practise that further reduces fruit load is known as thinning. The insufficient nature of physiological shedding of apple fruitlets can be improved by applying mechanical procedures or chemicals such as benzyladenine (BA), a synthetic cytokinin, that exerts its shedding action by promoting shoot growth which leads to competition for the nutrients of the tree. This nutrient competition ultimately influences the dominance between fruit clusters and between fruits of the same cluster [6,13–19]. Nutrient shortage produced by BA is supposedly first detected by the fruitlet cortex. Just two days after treatment with BA, transcriptomic and metabolic changes can be detected in the cortex of abscising fruits (AFs) compared to non-abscising ones (NAFs). Some of these metabolic changes include the accumulation of sucrose and the production of reactive oxygen species (ROS). Consistently with the abscission potential, genes putatively involved in the production of ROS, abscisic acid (ABA) and ethylene signaling and in cross-talk between these hormones, as well as genes characteristic of senescence, show increased expression in the cortex of AFs [3].

The molecular mechanisms that control and lead to early physiological fruitlet abscission are completely unknown although it has been reported that this process is under hormonal control. Whilst auxin and ethylene seem to play major roles [6,20–21], ABA, gibberellins (GA), cytokinins and jasmonates have also been reported to be involved [21–23].

As mentioned above, the signal for abscission is believed to first be detected by the cortex and subsequently transmitted to the seeds, leading to embryo abortion. Previous studies have shown that changes in the AF seed transcriptome appear later (around 4 days after BA treatment) than in the cortex. Consistent with embryo abortion and developmental arrest, genes specific for embryo and/or seed development are down-regulated in AFs compared to NAFs, and the latter are also more active metabolically [3]. It has been suggested that ethylene, by diffusion from the cortex, could be transporting the abscission signal to the seeds. Finally, there are also indications of greater ROS production in AFs that, together with the ethylene signalling coming from the cortex and the nutritional stress coming from the tree, would lead to metabolic deceleration thus diminishing or arresting auxin biosynthesis. The decrease in the flux of auxin will increase the sensitivity of the pre-formed abscission zone (AZ) to ethylene, activating the processes that will lead to the detachment of the fruitlet [3,6,24].

In this work, we have undertaken a comparative transcriptomic analysis of seeds from central fruits and seeds from lateral fruits sampled 20 days after petal fall (DAPF). At this stage, seeds already show transcriptomic signatures reflecting the abscission potential of the fruits. We provide the first transcriptome analysis of seeds belonging to lateral and central fruits. The comparison of the two transcriptomes sheds light on the role of the seed in fruitlet abscission [3].

## Material and Methods

### Plant material

Samples were collected in 2013 from an orchard of untreated adult apple trees (*Malus x domestica* ‘Golden Delicious/M9’) trained with standard horticultural practices at the experimental fields of the Istituto Agrario San Michele all’Adige (IASMA). Fruitlets were separated into two different populations, regardless of their abscission potential, based on position inside the fruit cluster. Samples of the two populations, central and mid-sized laterals (L2) (Fig. 1), were collected at defined time points (16, 18, 20 and 22 DAPF) among more than 20 trees randomly chosen from two columns inside the orchard. Central fruits are the biggest for each cluster, and L2 lateral fruits that theoretically would have an average abscission potential were selected (fruitlets between 8–9 mm of diameter). Fruits were dissected and seeds collected for both groups of samples, frozen in liquid nitrogen and stored at -80°C for the subsequent molecular applications.

No specific permissions were required for the field experiments, since they were done in collaboration with the owner institution, IASMA. Field studies did not involve endangered or protected species, but only commercial apple trees cv Golden Delicious.

### RNA isolation

RNA extraction was based on the method of [54]. 200 mg of seeds from different fruitlets were weighed per sample and ground in a pestle and mortar and the resulted powder was re-suspended in CTAB buffer plus beta-mercaptoethanol. RNA concentration and integrity was measured in a 2100 Bioanalyzer (Agilent Technologies, Palo Alto, CA, USA). All samples yielded an RNA integrity number (RIN) greater than 8.

### Illumina sequencing

2–4 μg of total RNA from each sample were used to make individual bar-code libraries using Illumina TruSeq RNA kits and sequenced using an Illumina HiSeq 2000 sequencer (single end, 50 bp; Illumina, San Diego, CA, USA). Quality control of the raw sequence data was done using FastQC (Babraham Bioinformatics).

Reads were aligned to whole genome sequences from the Malus x domestica genome v1.0 (www.rosaceae.org) and analyzed using the CLC Genomics Workbench (www.clcbio.com). RPKM (reads per kilobase per million) were considered as expression values and datasets were deposited publicly at GEO database (GSE62415). Three biological replicates of seeds dissected from central and lateral fruits were used. After normalization, Baggerley’s test was used for statistical analysis of samples. Genes with p-value < 0.05 and with a fold change >1.5 or <-1.5 were selected for gene ontology analysis.

### Annotation of the apple genome and statistical analysis of differential distribution of GO terms

All predicted gene models in the fully sequenced apple genome (v1.0) were downloaded from www.rosaceae.org and annotated by assigning their associated generic GO terms and Enzyme codes (EC) using the Blast2GO program [25]. This was based on homology to proteins from other species (as determined by BLAST), and integration of information about the occurrence of INTERPRO functional domains identified by INTERPROSCAN [26] and the KEGG EC biochemical pathways. Annotations were further expanded using ANNEX (Myhre, et al. 2006). The following settings were used: BLAST searches were conducted for each protein (BLASTX, nr database, HSP cut-off length 33, report 20 hits, maximum e-value 1E-10), followed by mapping and annotation (e-value hit filter 1E-10, annotation cut-off 55, GO weight 5, HSP-hit coverage cut-off 20). Note that a gene might have more than one distinct function and therefore might be annotated with more than one GO functional category or EC code. To have a broader overview of annotation of putative function, the resulting generic GO terms were mapped onto the corresponding Plant GO slim terms. We performed significance analyses of differential distributions of GO terms in comparisons between different subsets of genes to the entire complement of genes in the genome by means of Fisher’s exact test.

### qRT-PCR

Primers (S1 Dataset) were designed using Primer-BLAST (www.ncbi.nlm.nih.gov/tools/primer-blast/) on exon-exon spans when possible. *Ubiquitin* was used as a normalization gene. Relative expression of numbers of copies was calculated by the ΔΔCt method [27].

## Results

### Analysis of apple seed RNAseq data

We have used RNAseq in an effort to identify genes that are differentially expressed between central and lateral fruit seeds. Such an analysis could contribute to understanding the pathways and mechanisms involved in physiological shedding that occurs prior to exponential fruit growth. Samples of seeds for RNA extraction, deep-sequencing and subsequent transcriptomic analysis were taken at 20 DAPF from untreated trees (neither chemically nor mechanically thinned) from an orchard of the Golden Delicious cultivar.

A total of 112568528 and 116834022 reads (average length of 50 bp) were obtained from RNA of the seeds of central and lateral fruits (L2) respectively. A trimming procedure was applied to each sample in order to increase the number of reads mapping to the reference genome. After trimming the average read length was 41.8 bp. Each sample is represented by three biological replicates and has an average number of reads of 37522842 and 38944674 for central and lateral fruit seeds respectively. Consistently with previous studies [28], 64–67% of reads can be mapped to the apple reference genome for each sample, and around 66% of these mapped uniquely (Table 1).

**Table 1:** Summary of reads mapping.

Fruitlet type	Replicate	Total reads per sample	Total reads per biological condition	Reads mapped to apple reference	%	Reads uniquely mapped to reference	%
Central	1	32419019	112568528	21640191	66,75	14320596	66,18
	2	46118178		30628092	66,41	20369337	66,51
	3	34031331		22786521	66,96	15067791	66,13
Lateral	1	21522360	116834022	14326525	66,57	9494552	66,27
	2	59057754		37998113	64,34	25078566	66,00
	3	36253908		24348488	67,16	16144604	66,31
Average		38233758		25287988		16745908	

Of a total of 63541 genes present in the apple genome, our sequencing reads were assembled onto 35780 and 36224 genes for central and lateral fruit seeds respectively (genes with reads per kilobase per million (RPKM) ≥ 0.5). Analysis of the transcriptome of central fruit seeds reveals a large number of expressed genes annotated with GO terms related to metabolic processes as well as terms for cellular components such as the Golgi apparatus, ribosomes, the nucleus and the vacuole, together with several terms on catalytic activities (S3 Dataset). These terms are characteristic for a growing and developing organ, such as the fruit.

### Transcriptomic profiling reveals differences in the expression of genes related to specific functional categories between seeds from central and lateral fruits

A total of 17459 genes (representing around 28% of the total number of annotated genes in the *Malus x domestica* genome) are differentially expressed (DE) between the seeds of central and lateral fruitlets (fold change ≥ 1.5 or ≤ -1.5). Applying Baggerley’s test [29] for statistical significance, 1200 of these genes have a p-value ≤ 0.05. After correcting for False Discovery Rate (FDR) we obtained a list of 507 DE genes of which 470 are up-regulated (fold change ≥ 1.5; FDR corrected p-value ≤ 0.05) and only 37 are down-regulated (fold change ≤ -1.5; FDR corrected p-value ≤ 0.05) (S2 Dataset).

A statistical analysis of the functional categories that are differentially represented between the two types of seed samples was performed in order to identify transcriptomic signatures that could unravel processes potentially involved in fruit abscission. Initially, gene models encoded by the apple genome were annotated using GO terms by means of BLAST2GO [25] on the basis of homology and additional sources of evidence (e.g. protein functional domains, biochemical pathways). As a result, a total of 356581 generic GO terms were assigned to 46930 apple genes, representing a substantial fraction (73.86%) of the 63541 genes encoded by the apple genome. Each annotated gene has on average 5.61 associated GO terms and in order to obtain a broader overview of plant biological functions encoded by apple genes, the generic GO terms were collapsed into their corresponding parent GO term present in the Plant GO Slim subset [30] resulting in 327375 Plant GO Slim terms. We then used the annotation obtained to search for functional categories that were overrepresented among the sets of genes identified as differentially expressed between our samples. In the pool of 470 up-regulated genes, 42 GO terms corresponding to biological processes, 23 to molecular function and 8 to cellular components were found to be enriched (p-value ≤ 0.005).

Among the biological processes, many terms related to defence and stress responses were found, for example wounding, chitin, cold, heat, biotic and abiotic stimuli, water deprivation and osmotic stress (S4A Dataset). A GO term concerning the response to ROS (GO:0042542) is also enriched. Interestingly, relatively little enrichment related to metabolism, transport or hormone response (including ethylene biosynthesis) was noted. Nevertheless, four hormone-related GO terms are enriched. ‘Respiratory burst involved in defence response’ (GO:0002679) together with ‘S-methylmethionine cycle’ (GO:0033528) could imply an increase in the synthesis of ethylene in lateral fruit seeds. In addition, two genes, MDP0000735747 and MDP0000137705, annotated with the GO terms ‘Gibberellin catabolic process’ (Biological process; GO:0045487) and ‘C-19 gibberellin 2-beta-dioxygenase activity’ (Molecular function; GO:0052634) are up-regulated in lateral fruit seeds. These two genes are putative orthologs of *A*. *thaliana GA2OX6* (AT1G02400) and *GA2OX1* (AT1G78440), respectively, that encode gibberellin 2-oxidases. GA2OX oxidases form part of the catabolic pathway of this hormone and act on C19 GAs by inserting a 2β-hydroxy group that results in the inactivation of the hormone [31–32]. The term ‘Regulation of ABA mediated signalling pathway’ (GO:0009787) comprises 2 genes (MDP0000893203 and MDP0000231674) encoding type 2C protein phosphatases, the homologues of which in *A*. *thaliana* (*ABI2*) show activities as negative regulators of ABA signalling [33]. Moreover, although not belonging to this enriched GO term, *MdABR1* (MDP0000235028), a putative ortholog of *A*. *thaliana ABR1*, another negative regulator of the ABA response [34], seems to be significantly up-regulated in lateral fruit seeds compared to central ones.

Interestingly, the regulation of transcription, which includes transcription factors, is enriched. The up-regulated genes in lateral fruit seeds to which such GO terms have been assigned belong to different families of transcription factors (TFs) such as NAC, WRKY and Homeobox, and consistent with our results include a number of APETALA2/Ethylene responsive factors (AP2/ERF). Among them is MDP0000848905, an ERF involved in the ethylene signalling pathway and putative ortholog of *A*. *thaliana EBP* (*ERF72*) that has been suggested to be involved in programmed cell death and ROS accumulation [35]. Finally, MDP0000258562, a likely ortholog of *A*. *thaliana CEJ1/DEAR1* (Cooperatively regulated by Ethylene and Jasmonate 1), has been reported to be involved in the regulation of cell death after fungal infection [36].

Although fewer GO terms for molecular function and cellular component classes are enriched (S4B, S4C Dataset), there are some categories that reveal interesting signatures related to the abscission process. The two genes of GA catabolism (biological process; GO:0045487) appear to have confirmed dioxygenase activity (molecular function; GO:0052634) and therefore may be involved in the de-activation of this hormone. Four epimerases or epimerase-like activities have been listed in the term UDP-glucose 4-epimerase activity (GO:0003978). At least one ortholog in *A*. *thaliana*, *UGE5*, is induced by ABA [37], and in previous studies in apple at least one UDP-glucose 4-epimerase was up-regulated in the cortex of AFs [3]. Seven genes are classified as cysteine-type peptidases (GO:0008234) and three are involved in protein ubiquitination (GO:0051865), genes putatively involved in the machinery of protein degradation the activities of which could be interpreted as indicators of seed abortion.

There is also a set of GO terms (GO:0006012; GO:0005975; GO:0047936; GO:0046369; GO:0009225; GO:0009750; GO:0043617; GO:0008515) that imply active sugar signalling. Among them there are terms related to metabolism, transport and response to sugars, and further analysis would be necessary to establish the involvement of these sugars on abscission triggering or signalling.

As regards cellular components (S4C Dataset), the most enriched category is that of the nucleosome (GO:0000786) (found also as a Biological process; nucleosome assembly; GO:006334). The up-regulation of histones belonging to those terms would be indicative of changes in the transcriptional regulation in response to abscission.

Among the set of 37 down-regulated genes 12 GO terms for biological processes, 5 for molecular function and 1 for cellular components were found to be over-represented. Most biological processes involved the response to ions (calcium, lithium, zinc, manganese) or their homeostasis and transport (S5A Dataset). Interestingly, seven (out of 18) of the significantly enriched GO terms for down-regulated genes are specifically related to Ca^2+^ transport, homeostasis and response (GO:0015369; GO:008324; GO:0006874; GO:0006812; GO:0070588; GO:0006816). Ca^2+^ is important for enzyme function and it is known to be essential in several developmental processes including cell division and elongation, and fruit growth [38]. Ca^2+^ deficiency due to the down-regulation of genes encoding transporters of this cation could be a signal for the degeneration of the lateral fruits at this stage. In regard to the molecular function category, the two most significant GO terms concern cation-related transport (GO:0015369; GO:0008324) (S5B Dataset). Consistently in this respect, the only cellular component GO term for the down-regulated set of genes was for ‘plant-type vacuole membrane’ (S5C Dataset), the vacuole being the largest intracellular store of Ca^2+^ [39].

### Expression analysis of selected genes suggested to be involved in the abscission potential of lateral fruitlets

In order to validate our RNAseq data we performed qRT-PCR to check the expression of a subset of three genes known to be active in the developing seed and to be markers for fruitlet abscission [14,15,17]

**Fig 2:**
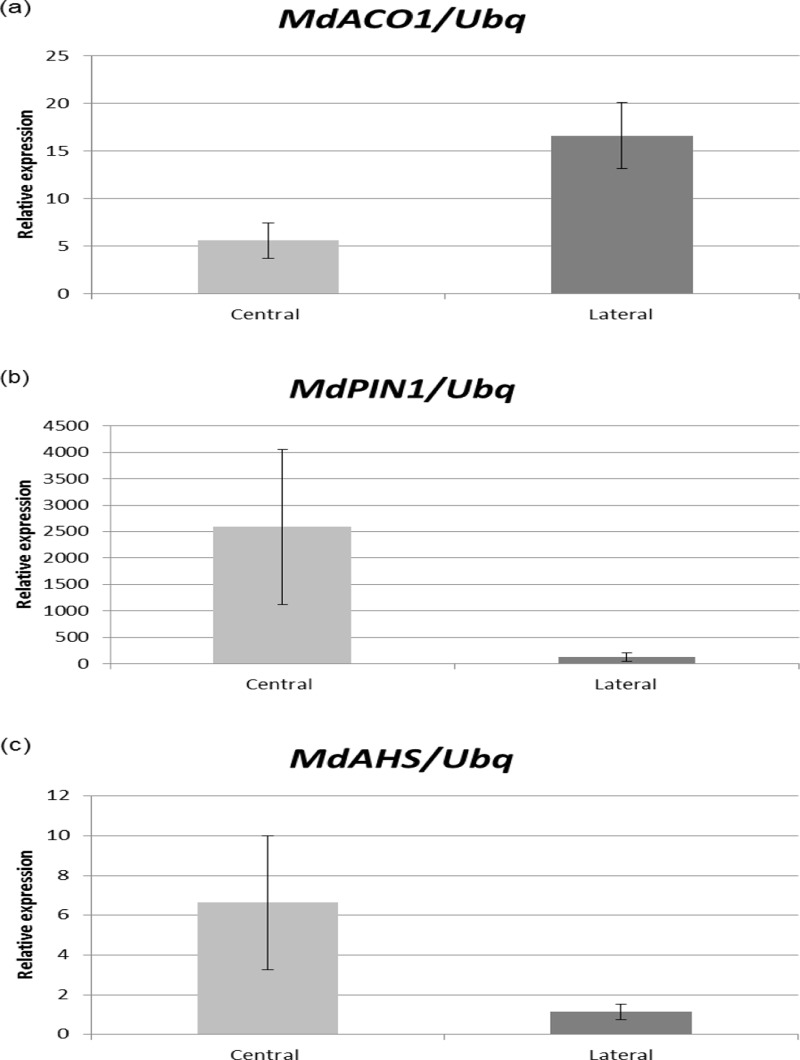
Gene expression at 20 DAPF. Comparison of expression of *MdACO1* (a), *MdPIN1* (b) and *MdAHS* (c) between apple central and lateral fruit seeds. Relative expressions in numbers of copies compared to the expression of the housekeeping gene encoding ubiquitin were obtained by qRT-PCR by means of the ΔΔCt method. Statistically significant differences were determined by Student’s *t*-test (*P* ≤ 0.05).

*1-aminocyclopropane-1-carboxylate oxidase1* (*MdACO1*; MDP0000195885) is the possible ortholog of the *A*. *thaliana* gene *ethylene-forming enzyme* (*EFE*; AT1G05010) which encodes a key enzyme in ethylene biosynthesis and one of the earliest markers of fruitlet abscission. Previous studies showed that *MdACO1* up-regulation is coupled to the peak of synthesis of ethylene in lateral fruits (between 19–21 DAPF depending on the size of fruits) and that both events (the synthesis of ethylene and *MdACO1* up-regulation) are consistent with a high abscission potential [3,14–16]. At 20 DAPF, *MdACO1* expression is greater in lateral fruit seeds than in central fruit seeds, indicating that the pathway for ethylene biosynthesis is activated in the seeds of lateral fruitlets (Fig. 2a).

The auxin:hydrogen symporter encoded by *MdAHS* (MDP0000154046; the putative ortholog of *A*. *thaliana* AT2G17500) and the auxin efflux carrier encoded by *MdPIN1* (MDP0000138035; the putative ortholog of *A*. *thaliana PIN1*) are good markers for PAT in fruitlets. Most auxin biosynthesis in the fruit takes place in the seeds, so measuring auxin transporter gene expression in seeds should be a good indicator of PAT flux. When measured at 20 DAPF both *MdAHS* and *MdPIN1* expression is very reduced in lateral fruits compared to central fruits, suggesting that these fruits have reduced PAT (Fig. 2b). A reduction in auxin transport would result in an increase in the sensitivity to ethylene at the abscission zone (AZ) thus triggering the process of detachment of the fruitlet [6,40].

The results of the expression analysis of these three genes support the validity of our RNAseq and are consistent with the high abscission potential of the lateral fruits that were selected.

### The dynamics of genes involved in hormonal signalling

Previous studies have highlighted the role of the seed as the detector of the abscission signal, triggered at the cortex possibly due to nutritional shortage, and putatively translated to the seed by hormones, mainly ethylene [3]. In order to assess the molecular response of genes involved in hormonal signalling in both types of seeds (from central and lateral fruitlets), a four point time-course of the expression of a selected subset of genes has been performed with a view to identifying differences that could help explain the role of the seed in the different abscission potentials of lateral fruits. qRT-PCR was performed on RNA extracted from lateral and central fruit seeds. All data are presented in terms of relative expression compared to the reference gene.

**Fig 3:**
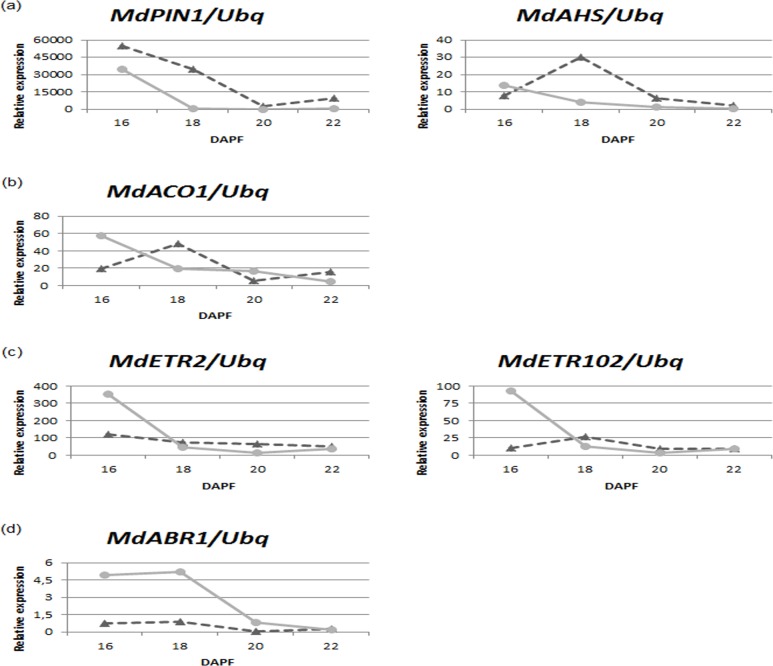
Dynamics of expression of apple genes. Comparison between apple central (triangles and dotted lines) and lateral fruit seeds (circles and continuous lines) of dynamics of expression of genes involved in the biosynthesis, transport or response to different hormones. (a) *MdPIN1* and *MdAHS*, involved in auxin polar transport (PAT). (b) *MdACO1*, involved in ethylene biosynthesis. (c) *MdETR2* and *MdETR102*, involved in response to ethylene. Relative expression was obtained by qRT-PCR by means of the ΔΔCt method compared to the expression of the housekeeping gene encoding ubiquitin.

Overall, both the *MdAHS* and *MdPIN1* genes present expression dynamics indicative of greater auxin transport in the seeds of central fruits (Fig. 3a). The kinetics of *MdPIN1* are interesting as they suggest that auxin transport decreases over the period analysed, but the main point of note is that the levels of expression of both auxin transporters in central fruit seeds are much higher than those in lateral fruit seeds, consistent with greater auxin biosynthesis in the former. In addition, *MdPIN1* decreases its expression in lateral fruit seeds from 18 DAPF almost to basal levels and this should promote the disruption of PAT towards the pedicel.

In this work we have used *MdACO1* expression as a proxy for the biosynthesis of ethylene over this period. Our data show that at 16 DAPF the expression of *MdACO1* is much higher in lateral compared to central fruit seeds, but that this is rapidly down-regulated from 18 DAPF and that these levels are subsequently maintained until 22 DAPF (Fig. 3b). In addition, a peak of *MdACO1* expression is observed in central fruit seeds at 18 DAPF which then returned to basal levels between 20 and 22 DAPF (Fig. 3b). In previous studies by contrast, it has been noted that basal levels of ethylene are synthesized in both central and lateral fruitlets, although these levels are somewhat higher in the latter with a peak occurring between 19 and 21 DAPF depending on the dimensions of the fruit (L3 and L1 respectively) [3]. These differences may be accounted for by the fact that we are monitoring ethylene synthesis solely by the seed.

The expression dynamics of two ethylene perception genes belonging to ethylene receptor subfamily 2 were also assessed. *MdETR2* (MDP0000219737) and *MdETR102* (MDP0000920189) are likely orthologs of *A*. *thaliana ETR2* and both are strongly induced after ethylene treatment [41]. In lateral fruit seeds, the levels of expression of both genes are consistent with those of *MdACO1* and thus with ethylene biosynthesis. At 16 DAPF both *MdETR2* and *MdETR102* are highly expressed in lateral fruit seeds compared to those of central fruits (the latter maintain a stable level of expression from 18 to 22 DAPF). From 18 DAPF both genes are down-regulated in lateral fruit seeds and their expression is lower than that in central fruit seeds from 18 to 22 DAPF (Fig. 3c).

Finally, we checked the dynamics of *MdABR1* expression, a putative negative regulator of the ABA response (Fig. 3d). We found a 5-fold up-regulation from 16 to 18 DAPF in lateral fruit seeds compared to those of central fruits, and a strong decrease from 20 DAPF onwards.

Our results suggest that PAT is compromised or at least very reduced in lateral fruits. Ethylene biosynthesis is greater at 16 DAPF in lateral fruits which is consistent with the high levels of expression of two negative regulators of the ethylene response. Moreover, we found a similar expression pattern for *MdABR1*, a negative regulator of the ABA response. Taken together these data suggest the concerted expression of a set of genes in an attempt to maintain the homeostasis of the seed against hormones that promote abscission and the disruption of PAT.

## Discussion

To the best of our knowledge, the present study is the first deep-sequencing analysis of apple seeds focused on fruit abscission. Previous work has shown that transcriptomic changes at seed level could be detected 4 days after a thinning treatment with BA to increase apple fruit physiological drop [3]. In our work we have analysed global gene expression in seeds collected from the central and lateral fruits of untreated trees with a view to elucidating the molecular mechanisms and biological pathways involved in physiological abscission, as well as assessing the role of seeds in this process.

### Hormone-related transcriptomic signatures in apple seeds from lateral fruitlets

RNAseq was chosen not only because of the availability of the apple genome [1] which allowed us to assign more than 65% of reads to already annotated genes (Table 1), but also because it facilitates the discovery of new genes and the generation of transcriptomic profiles (S3 Dataset). Importantly, 35% of reads remained unassigned in our study (not assembled) which opens the possibility of performing *de-novo* analysis for the mining of new genes in the apple genome. The validity of the RNAseq data was assessed by qRT-PCR (Fig. 2) and this was also used to determine the abscission potential of lateral fruitlets. In general terms, we detected transcriptomic changes that suggest the triggering of responses to different hormonal signals in the seeds of L2 fruitlets consistent with a high abscission potential.

One of the most studied features of fruitlet abscission is the influence of PAT. Auxin is mainly produced in seeds, although lower levels are also synthesized in the cortex [42]. Our data on the expression of auxin transporters (Fig. 3a) suggest that PAT is notably reduced in lateral fruitlets, consistent with a high abscission potential.

GAs are hormones involved in cell expansion, fruit set and growth [43,44]. Previous studies have demonstrated the expression of *GA20OX* genes in developing apple seeds and are hence indicative of active GAs biosynthesis [45]. However, we have found an activation of GA catabolism in lateral fruit seeds that would lead to a decrease in bioactive GAs. The expression of *GA2OX* genes has also been described in the cortex of lateral fruits having a high abscission potential [3]. This is the first time that expression of *GA2OX* has been described in seeds during fruit abscission. Up-regulation of these genes in lateral fruit seeds might suggest that the signal for abscission has already been triggered and therefore that seed development has stopped.

The importance of ethylene biosynthesis in the apple fruitlet abscission process has been demonstrated for both signalling at fruit level and for triggering separation at the AZ [3,21,46,47]. Whilst no GO term directly concerning the biosynthesis of ethylene has been found, a related term, ‘S-methylmethionine cycle’ (GO:0033528), annotating two up-regulated genes was found to be significantly enriched. Methionine has been shown to be a precursor of ethylene in different species including apple [48,49].

Although the GO term ‘ethylene biosynthetic process’ was not significantly enriched it includes 4 genes significantly up-regulated in lateral fruit seeds. Among them is *MdACO1* (MDP0000195885), a key enzyme in the biosynthesis of ethylene and one of the earliest markers of fruitlet abscission [3,14–16]. Our results on the dynamics of *MdACO1* expression in lateral fruit seeds during young fruit development (Fig. 3b) suggest that ethylene biosynthesis in this organ is uncoupled from its biosynthesis in the cortex as no peak of ethylene has been found at 16 DAPF when whole fruits are taken into consideration [3]. In whole fruits (seed plus cortex) the ethylene peak and the elevated expression of *MdACO1* concur at 21 DAPF [3]. The biosynthesis of ethylene in seeds could be masked by dilution when measured in whole fruits, whereas our results point towards specific ethylene production within the seed albeit at lower levels with respect to those in the cortex.

The dynamics of the expression of *MdETR2* and *MdETR102*, two ethylene receptors, seem to be coupled to ethylene biosynthesis in lateral fruit seeds and correlate with the expression of *MdACO1* (Fig. 3b,c). As ETRs are negative regulators of the ethylene response [50], we speculate that their co-expression with *MdACO1* may act as a protection mechanism to maintain seed homeostasis.

In this regard, a set of 6 genes included in the GO term ‘regulation of transcription‘ (GO:0006355) belong to the ERF family of TFs, part of the AP2/ERF superfamily. Some of them have been related to stress responses involved in protection against cell death [35,36]. Their up-regulation in lateral fruit seeds further supports the contention that the ethylene response is activated. Nevertheless, we cannot exclude the possibility that the high expression levels of some ETRs in the seed are a response to ethylene produced in the cortex, as these same receptors do not respond to local ethylene production in central fruit seeds (Fig. 3b,c).

ABA has been related to stress-induced senescence [51] and is known to have a stimulatory effect on abscission. However whether this is a direct effect or acts via stimulation of the biosynthesis of ethylene is still controversial [22,47]. In some species ABA is known to stimulate fruit and leaf abscission in a concentration-dependent manner [52,53]. In lateral fruit seeds at least three up-regulated genes with putative activities as negative regulators of the ABA response have been found. *MdABR1* expression during fruitlet development is greater in lateral fruit seeds compared to those of central fruits (Fig. 3d). Moreover, elevated expression of other negative regulators of ABA signalling has been found in lateral fruit seeds. Similarly to the case of the ethylene response, we speculate that the over-expression of negative regulators of the ABA response could represent a mechanism of protection against strong abscission hormonal signals which can be produced by the cortex or locally in the seed. At a certain point the signal overcomes these mechanisms, manifested as a down-regulation of gene expression during seed development and eventually leading to the abscission of the fruit.

### The putative role of seeds in the model of apple fruitlet abscission

According to our results, at 20 DAPF seeds from lateral fruitlets present transcriptomic signatures consistent with a high abscission potential. Indeed, our transcriptomic analysis confirms previous studies [3] that showed that several transcriptomic signatures can be observed in seeds from 4 days after inducing abscission with BA (applied at 15 DAPF). In our study, we have compared the seeds of central and lateral fruits at 20 DAPF from untreated trees and we detected several signatures confirming the process of physiological abscission in lateral fruits. RNAseq has also enabled us to find new genes and pathways involved in the abscission process. Our GO enrichment analysis for the set of up-regulated genes has shown a vast number of terms related to stress and defence responses (S4A Dataset). This feature is typical of lateral fruits regardless of their abscission potential. The enrichment of genes involved in ubiquitination (GO:0051865) and in peptidase activity (GO:0008234) could be indicative of activation of protein degradation and hence degeneration of the seed. In this sense, the down-regulation of Ca^2+^ transport and homeostasis related genes could also be an indicator of a decrease in metabolic rate, consistent with seed developmental arrest.

With respect to hormonal signalling, various signatures are present in our analysis. The activation of GA catabolism and the putative disruption of PAT suggest the arrest of seed development. Ethylene biosynthesis seems to be activated, but its levels in seeds may be much lower than those in the cortex, and it is still unknown if this biosynthesis is related to the abscission process.

## Conclusions

Our seed transcriptomic data suggest that lateral fruits are ‘pre-conditioned’ to abscise, and that the effect of blooming time and fertilization as well as position inside the corymb are important for conditioning the transcriptome of the seed, leading to specific features that will ultimately decide the fate of the fruitlet according to the nutritional conditions of the plant. These seeds are thus ‘prepared’ for the stimulus and rapidly respond to it by triggering the hormonal response leading to developmental arrest. We cannot exclude the influence of developmental stage between central and lateral fruits in the differences observed. The latter are younger and this might explain some of the differences in the expression levels of the genes tested. Nevertheless, this is the physiological situation inside the corymb. Central fruit fertilization and set can condition the lateral fruits, which physiologically will have reduced PAT and more ethylene. From our results, we can suggest that most of the lateral fruits are already conditioned to abscise. This conditioning could putatively be a protection mechanism to save the most adapted fruit, the central fruits, in case of nutritional or environmental stress. Seeds from lateral fruits seem to be activating a mechanism to maintain homeostasis and avoid abscission, and eventually, depending on their position in the cluster, they may be unable to avoid triggering the signal that will lead to developmental arrest and eventually abscission. As regards the abscission process, seeds would play a role in stopping physiological shedding. It remains to be elucidated whether they, or the cortex as has been previously proposed [3], are also the primary sensors of the abscission signal as we cannot exclude the possibility of the seed-specific early peak of ethylene biosynthesis being the signal for developmental arrest. In order to establish a more precise model and identify the abscission signal trigger, further studies are necessary to determine both the nature of the hormonal signalling before 15 DAPF in these fruits and the cross-talk between the cortex and the seed. Nevertheless, our study represents an important step for elucidating the biological pathways involved in young fruit abscission, and more studies are being conducted to unravel new genes and functions involved in this process.

## Supporting Information

S1 Dataset Apple gene specific primers for qRT-PCR. (XLSX)

S2 Dataset Differentially expressed (DE) genes between central and lateral fruit seeds. Only statistically significant genes are shown with FDR *P-value* correction ≤ 0,05.
(XLSX)

S3 Dataset GO terms associated with the complete transcriptome of central fruit seeds. Only terms with more than 10 genes associated are shown, compared to the whole set of annotated apple genes for each term.
(XLSX)

S4 Dataset GO terms associated with the set of up-regulated genes in lateral fruit seeds. Worksheet: (3A) Biological process; (3B) Molecular function; (3C) Cellular component. *P-values* were calculated by Fisher’s exact test.
(XLSX)

S5 Dataset GO terms associated with the set of down-regulated genes in lateral fruit seeds. Worksheet: (4A) Biological process; (4B) Molecular function; (4C) Cellular component. *P-values* were calculated by Fisher’s exact test.
(XLSX)
